# Satellite-Enabled Internet of Remote Things Network Transmits Field Data from the Most Remote Areas of the Tibetan Plateau

**DOI:** 10.3390/s22103713

**Published:** 2022-05-13

**Authors:** Yingying Chen, Minghu Zhang, Xin Li, Tao Che, Rui Jin, Jianwen Guo, Wei Yang, Baosheng An, Xiaowei Nie

**Affiliations:** 1 National Tibetan Plateau Data Center, State Key Laboratory of Tibetan Plateau Earth System, Resources and Environment (TPESRE), Institute of Tibetan Plateau Research, Chinese Academy of Sciences, Beijing 100101, China; chenyy@itpcas.ac.cn (Y.C.); xinli@itpcas.ac.cn (X.L.); yangww@itpcas.ac.cn (W.Y.); anbaosheng@itpcas.ac.cn (B.A.); xwnie@itpcas.ac.cn (X.N.); 2 CAS Center for Excellence in Tibetan Plateau Earth Sciences, Beijing 100101, China; 3 School of Computer and Communication, Lanzhou University of Technology, Lanzhou 730050, China; 4 Heihe Remote Sensing Experimental Research Station, Key Laboratory of Remote Sensing of Gansu Province, Northwest Institute of Eco-Environment and Resources, Chinese Academy of Sciences, Lanzhou 730000, China; chetao@lzb.ac.cn (T.C.); jinrui@lzb.ac.cn (R.J.); guojw@lzb.ac.cn (J.G.)

**Keywords:** satellite-enabled Internet of Remote Things, Tibetan Plateau (TP), extreme area, remote data retrieval

## Abstract

In this article, we employed a satellite-enabled Internet of Remote Things (IoRT) network as a promising solution to retrieve data in the most remote areas of interest, where public networks are absent. This article presents a system network based on the satellite-enabled IoRT, a new paradigm that defines a network where each environmental monitoring device can autonomously establish a network with a remote data center. The Xingyun satellite constellation was employed for data retrieval on the Tibetan Plateau (TP). The monitoring system was mainly composed of a ground Internet of Things (IoT) terminal that was built with satellite transceivers, environmental monitoring devices, and system software. We deployed five of these newly developed terminals in harsh areas to monitor environmental variables, and accordingly, air temperature and relative humidity, precipitation, snow depth, land surface temperature, tree stemflow rate, and photosynthetically active radiation were retrieved with the satellite-enabled IoRT network. Field experiments were conducted to evaluate the performance of the proposed system network, and the results indicated that the average time delay with and without the packet creation mode reached 32 and 32.7 s, respectively, and the average packet loss rate with and without the packet creation mode reached 5.63% and 4.48%, respectively. The successful implementation of the satellite-enabled IoRT network for the rapid retrieval of monitoring data in remote glacier, forestland, and canyon areas at very high altitudes on the TP provides an entirely new and revolutionary data retrieval means for backhauling data from remote areas of interest.

## 1. Introduction

The Tibetan Plateau (TP) is the source of 10 major Asian rivers and covers an area of 2.5 million km^2^ with an average elevation exceeding 4000 m above sea level (a.s.l.), and thus, this region is also referred to as the Asian Water Tower, Roof of the World, and Third Pole of the Earth [1,2,3,4]. Over the past half century, the warming rate on the TP has reached twice the global average, leading to dramatic environmental changes [5], such as rapid lake expansion [6], steadfast glacier retreat [7,8], and even glacial collapse in recent years [9]. However, the scarcity of observation data due to harsh environmental conditions has impeded scientists from answering scientific questions regarding hydrological, ecophysiological, and biogeochemical interactions on the TP [10,11]. Large-scale deployment of automatic ground-based observation stations provides the potential to revolutionize the way we understand multisphere interactions and human-nature relationships across the TP. However, the retrieval of monitoring data from ground stations deployed in the farmost areas of interest in extreme environments remains a great challenge.

To provide faster and more reliable data collection, most ground stations are deployed close to residential areas to facilitate maintenance and rapid data transmission. Monitoring data can be retrieved through public communication networks. However, observation facilities have been installed close to glaciers, ice lakes, remote forests, and other farmost areas, where the public communication networks are absent and monitoring data are retrieved manually one or two times per year. Thus, to realize the rapid retrieval of monitoring data throughout the farmost areas without public networks, the state-of-the-art Internet of Things (IoT) [12,13] is essential. In this paper, we proposed an IoT architecture allowing the retrieval of observation data in the farmost areas on the TP.

The proliferation of the IoT and its applications in environmental monitoring have enabled the deployment of intelligent monitoring devices in remote areas of interest for real-time environmental sensing [14,15]. Researchers have illustrated cases of how the IoT can be employed in extreme environments, such as the Arctic or Antarctic [16,17]. However, none of these previous studies explored the exploitation of the satellite-enabled Internet of Remote Things (IoRT) to retrieve data at the farmost Third Pole of the Earth, i.e., the TP. Furthermore, the introduction of a satellite-enabled IoRT in extreme areas has become the only effective way to enhance the operational efficiency of data retrieval and to decrease device setup and data collection costs [18,19]. In this article, we propose a novel data retrieval framework by using China’s first IoT satellite constellation, namely, the Xingyun project, with the goal of transmitting in situ monitoring data from the farmost areas of the TP. We developed a ground IoT terminal device comprising an environmental monitoring device and satellite-based transceiver to rapidly achieve data backhaul. Environmental monitoring devices, such as weather stations, were deployed in the area of interest to monitor the surrounding environment. A satellite-enabled IoRT technique, which could resolve the lack of data on the TP, was established while reducing the data backhaul time and cost in the farmost areas without public networks.

To verify the effectiveness of the satellite-enabled IoRT network in regard to field data transmission from the farmost areas of the TP where public networks are absent, five field experiments were conducted on the TP, which exhibited significance for scientific research. The contributions of this paper can be summarized as follows:

- A satellite-enabled IoRT network integrating IoT satellites, environmental monitoring devices, and sensors was utilized to retrieve in situ data from the farmost areas of interest on the TP.
- Application of the proposed ground IoT terminal device was demonstrated on the TP, illustrating that the proposed method could efficiently transfer monitoring data to a data center at an increased performance and lower cost.
- Experiments were conducted to reveal the efficiency of the satellite-enabled IoRT network. We analyzed the results and demonstrated the value of the IoRT network in environmental field studies.

## 2. Related Work

Environmental monitoring in the farmost areas is becoming increasingly desirable largely because many studies have noted the importance of understanding environmental processes and their effect on climate change. Thus, without in situ and long-term monitoring data, the determination of the fingerprints of complex interactions becomes increasingly difficult. Data retrieval in the farmost areas without public networks is one of the difficulties referenced in previous studies.

### 2.1. TP Monitoring

Considering the crucial impact of the TP, scientists have recognized the great value of observation data on the TP and the implications for scientific research. Over the last several decades, a number of comprehensive observation stations have been built on the TP by Chinese research institutions. The Institute of Tibetan Plateau Research Chinese Academy of Sciences allied 21 stations to form the Tibetan Observation Network and Research Platform (TORP) [20] with the aim of providing long-term and in situ monitoring data. In 2013, the High-cold region Observation and Research Network for land surface processes and environment of China (HORN) was officially established to provide long-term and stable support to 17 comprehensive observation and research stations across the TP, and most TORP stations were included. Along with the ongoing advances in wireless technologies, several large-scale scientific experiments, such as the Global Energy and Water Cycle Experiment (GEWEX), Asian Monsoon Experiment on the Tibetan Plateau (GAME/Tibet, 1996–2000) [21], Coordinated Enhanced Observing Period (CEOP) Asia-Australia Monsoon Project on the Tibetan Plateau (CAMP/Tibet, 2001-06), Heihe Watershed Allied Telemetry Experimental Research (HiWATER) [22], and Third Tibetan Plateau Atmospheric Scientific Experiment (TIPEX-III) [23,24] have been conducted in recent decades and have significantly enhanced the Earth system observations and research on the Earth science system of the TP.

While the ground observation network plays an important role in scientific research, these systems cannot retrieve data in the farmost areas of the TP due to the high construction cost and notable dependence on public networks. Thus, remote sensing has drawn much research attention regarding effective monitoring in inaccessible areas where in situ observations are absent [25,26]. However, the high uncertainty in satellite-retrieved environmental variables has resulted in monitoring data discontinuities on a temporal–spatial scale. Additionally, considering the sparsity of observation stations and uncertainty in remote sensing monitoring, researchers [27,28] have obtained environmental data in the farmost areas of the TP via interpolation and integrated learning methods. Although scientists have made great efforts to mitigate the problem of insufficient observation data on the TP, none of the presented works addressed observation data retrieval requirements in the farmost areas on the TP.

### 2.2. Remote Data Retrieval

The IoRT network, which is highlighted for enabling the deployment of IoT devices across wide geographical areas, could retrieve monitoring data in the farmost areas of the TP [29,30]. The IoRT network can be defined as the art and science of using advanced technology, such as unmanned ships, drones, and satellites, to transmit in situ data cached in ground IoT terminals, deployed in the field for environmental monitoring [31,32,33]. In general, the ground IoT terminal is an integrated device comprising a datalogger, sensors, and a communication module with a transceiver antenna for data transmission and receival. Additionally, in the satellite-enabled IoRT, a ground gateway station is required to transfer the collected data from satellites to scientific data centers via the Internet. As a platform, the data center is used for observation data publishing.

Data retrieval in the farmost areas is being revolutionized by the IoRT [34,35]. Numerous IoRT applications have been demonstrated under different scenarios (e.g., environmental monitoring, beach monitoring, and ice sheet monitoring), where public networks are absent [36,37]. For example, a drone-based wireless sensor network (WSN) was introduced to establish an IoRT network for marine coastal monitoring [38]. According to this study, a drone was employed as a mobile relay to collect data from sensing buoys. In addition to marine coastal monitoring, the authors of [39] proposed a drone-based IoRT network for crop monitoring. The proposed network, which comprised a WSN and aerial robot, was used for frost monitoring in vineyards. The established network could overcome the limitation of transmitting monitoring data via a public network.

Moreover, Sousa et al. examined the remote data backhaul process from another perspective, thus proposing an unmanned vehicle-based IoRT network via autonomous underwater vehicles for low-cost oceanographic and environmental surveys [40]. Similarly, Delphin et al. combined different acoustic navigation and underwater vehicles for Arctic under-ice monitoring [41]. In addition to the problem of retrieving data in areas without public networks, many studies have been conducted with the aim of improving the data retrieval efficiency via novel and efficient satellite-enabled IoRT networks. Accordingly, the authors of [42] proposed a satellite communication-aid network to transmit power system operation monitoring data, which indicated that satellite communications could be suitably used to transmit SCADA/EMS data in theory. Similarly, as stated in [43], a satellite-based system was presented for remote animal behavior monitoring in the wild. These studies did not verify the ability of remote data transmission using satellite-enabled IoRT networks from a practical application perspective. Thus, motivated by the above works, we are interested in exploiting the IoRT using satellites, which could boost the data retrieval performance in remote areas.

## 3. Architecture of the Satellite-Enabled IoRT Network

**Figure 1:**
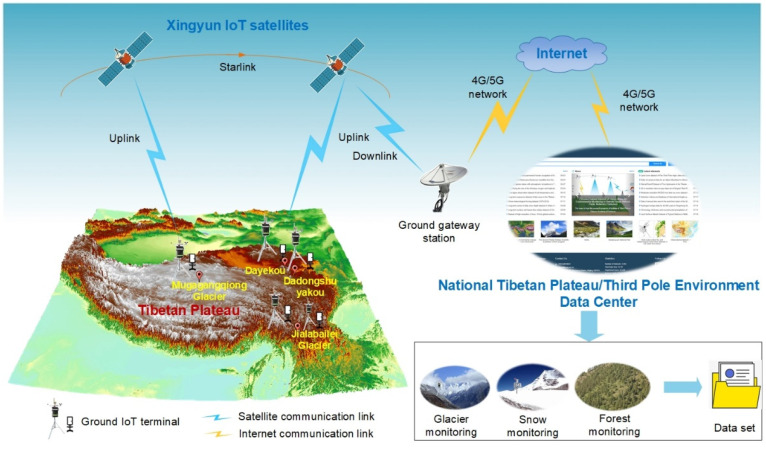
Implementation architecture of the Xingyun satellite-enabled Internet of Remote Things network for data retrieval from the Tibetan Plateau (TP). Five satellite-enabled ground IoT terminal devices are installed in the far-most areas of the TP, i.e., the Mugagangqiong glacier area, Jialabalei glacier area, Dadongshuyakou snow-covered area, and Dayekou forest. These devices monitor the local environment and upload remotely sensed data to IoT satellites during overpass. The satellites then transmit the uploaded data to the TP scientific data center via a gateway station and the Internet. The scientific data center finally publishes a monitoring dataset on an open-access web platform.

Because satellites can acquire observations with global coverage under all weather conditions, satellite-based IoRT networks could revolutionize the rapid retrieval of in situ data in remote areas of interest. The proposed IoRT network comprises three main parts: ground IoT terminals, an IoT satellite constellation, and a data center. The physical architecture of the proposed satellite-enabled IoRT network is depicted in Figure 1.

(1) Ground IoT terminals, each of which comprises monitoring devices and a satellite-based transceiver, are installed in remote areas of interest for real-time monitoring and rapid data transmission. A traditional environmental observation device is employed to monitor the environment, and the monitoring data are transmitted via a satellite-based transceiver. Notably, these ground IoT terminals can integrate a diverse range of monitoring devices, such as automatic weather stations (AWSs).

(2) China’s first IoT satellite constellation is used to establish the satellite-based IoRT network for field data transmission from the ground IoT terminals to the data center. The satellite communication links include an uplink from the ground IoT terminal to a satellite, an intersatellite link within the satellite constellation, and a downlink from the satellite to the data center. The monitoring data cached in the ground IoT terminals are transferred to the IoT satellite via uplink and then transferred to the data center via a ground gateway station and the Internet.

(3) A data center is established as a platform to receive, manage, analyze, and publish the monitoring data obtained from the most remote areas of the TP.

### 3.1. IoT Satellite Constellation

A key aspect of the satellite-enabled IoRT network is remote data transmission via satellites. China’s first IoT satellite constellation has greatly motivated this study of the IoRT for in situ data transmission from remote areas. The Xingyun satellites can encourage stakeholders to support remote monitoring in remote areas across the TP. The satellites of the Xingyun IoT satellite constellation communicate in the L frequency band, and these satellites can transmit sensor data and realize bidirectional short-message communication. Hence, the satellite-enabled IoRT can facilitate data retrieval in remote areas arbitrarily yet efficiently.

At present, the two Xingyun satellites pass over the same observation station 4 times a day (2 times per satellite), with each pass lasting 5–10 min. In addition, the Xingyun satellites are equipped with an intersatellite laser communication capability, which can reduce the downlink time. These characteristics enable rapid monitoring data collection.

### 3.2. Monitoring Devices Integrated with a LEOBIT Satellite Transceiver

**Figure 2:**
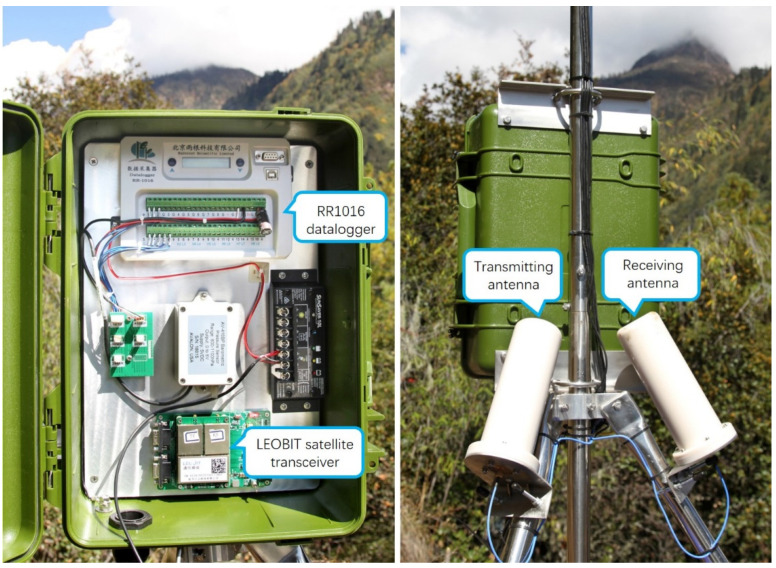
Prototype of the ground IoT terminal device for the Xingyun satellite IoT. The key components of the terminal device include a LEOBIT satellite transceiver, transmitting and receiving antennas, and RR1016 datalogger.

We developed a ground IoT terminal device for environmental monitoring based on the Xingyun satellite communication protocol. The device could forward monitoring data to the Xingyun IoT satellites during overpass across the site. Figure 2 shows a prototype of the ground IoT terminal device. The developed device included a satellite transceiver and datalogger. The operational frequency of the uplink communication ranged from 1668 to 1675 MHz, and the downlink receiving frequency ranged from 1518 to 1525 MHz. The downlink transmission rate of the transceiver was 2.4 kbps, and the uplink transmission rate was adjustable at 1.2, 2.4, and 4.8 to 9.6 kbps, which satisfied the requirements of conventional monitoring data transmission. Moreover, the satellite transceiver was a low-power device: the transmitting power reached approximately 1 W, and the average power consumption of the whole device was lower than 1.5 W. Table 1 summarizes the power consumption characteristics of the LEOBIT satellite transceiver under different working scenarios.

**Figure 3:**
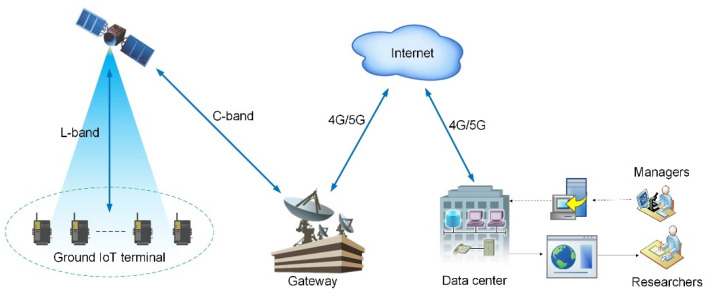
Interaction of diagram of the satellite-enabled IoRT system.

Additionally, the employed datalogger was a multichannel universal data collector that was specifically designed to automatically acquire observations in harsh environments based on the power supply excitation channel of the sensor. These characteristics allowed the datalogger to flexibly control the connection time and greatly reduce the system power consumption. Thus, the datalogger could operate with less than 1 W of power supplied by solar panels and was highly suitable for field deployment applications. In the prototype of the ground IoT terminal device for the Xingyun satellite IoT, the key components of the terminal device included a LEOBIT satellite transceiver, transmitting and receiving antennas, and RR1016 datalogger. The interaction between the data center and in situ sensors is shown in Figure 3.

### 3.3. Data Management System

**Figure 4:**
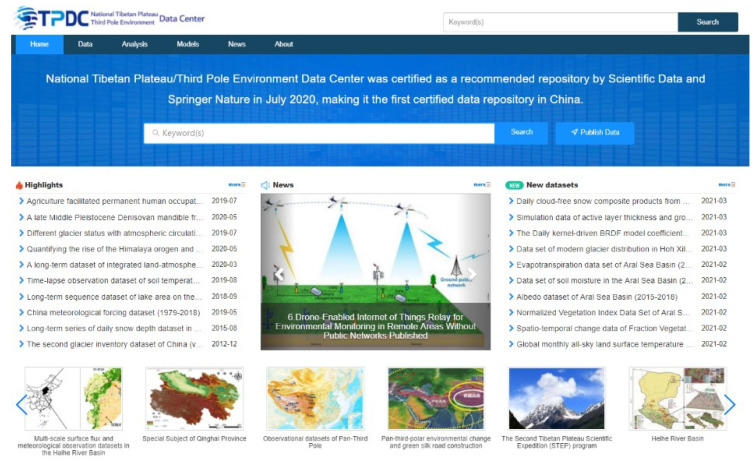
National Tibetan Plateau/Third Pole Environment Data Center.

The data management system [44,45], as presented in Figure 4, was implemented to receive, manage, and publish the monitoring data obtained on the TP via the satellite-enabled IoRT network. In this research, the National Tibetan Plateau/Third Pole Environment Data Center (TPDC) functioned as the data management system of the proposed satellite-based IoRT network. The TPDC is one of the first 20 national data centers approved by the Ministry of Science and Technology and the Ministry of Finance of the People’s Republic of China in 2019. The TPDC is the only data center in China that stores comprehensive scientific data pertaining to the TP and surrounding regions. As of May 2021, the TPDC hosts more than 3500 datasets. A cloud service platform was developed at the TPDC to facilitate the extensive integration of data, methods, models, and services with the goal of enabling big data analysis for TP research. The TPDC was certified as a recommended repository by Scientific Data and Springer Nature in July 2020, making it the first certified data repository in China.

## 4. Demonstrated Application and Analysis

### 4.1. Demonstrated Application

**Figure 5:**
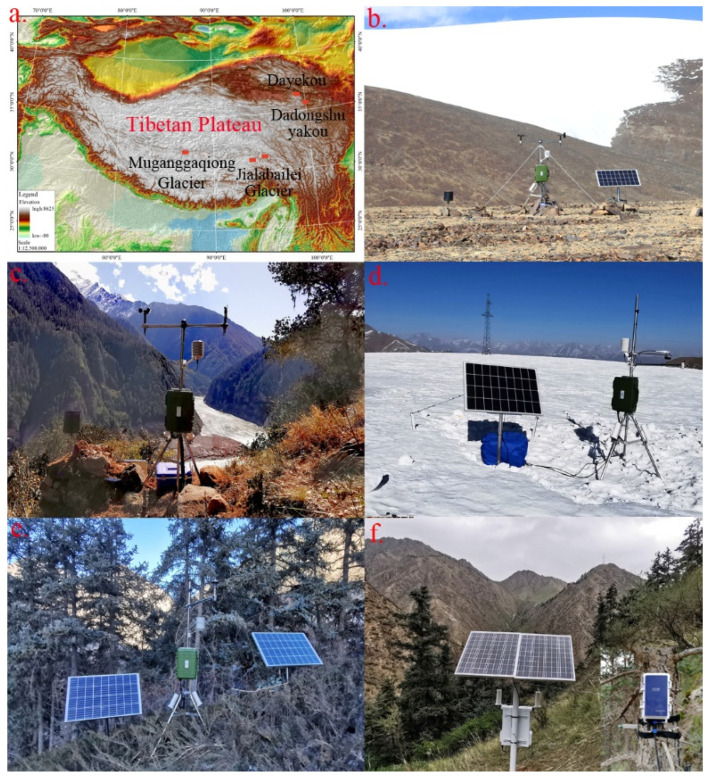
Locations of the five ground IoT terminal devices and observation facilities in the far-most area of the TP (**a**) and photos of these installations at the end of the Mugagangqiong glacier (N 32.220°, E 87.485°, 5706 m.a.s.l.) (**b**), at the dammed point on the Yarlung Zangbo River below the Jialabailei glacier (N 29.748°, E 94.934°, 3033 m.a.s.l.) (**c**), in the Dadongshuyakou snow-covered area (N 38.015°, E 100.244°, 4174 m.a.s.l.) (**d**), and at two sites in the Dayekou forest area (N 38.580°, E 100.292°, 2564 m.a.s.l.) (**e**,**f**).

Five ground IoT terminal devices were installed in the most remote regions of the TP without a public communication network, as shown in Figure 5. On 23 October 2019, the first terminal device and AWS were installed at the end of the Mugagangqiong glacier to collect meteorological data. This glacier is situated in the hinterland of the Qiangtang Plateau, which covers an area of approximately 708,000 km^2^ with an average altitude ranging from 4700–5200 m and is referred to as the roof of the TP, which in turn is referred to as the Roof of the World. On 28 October 2019, the second terminal device and an AWS were installed on the slope opposite the Jialabailei glacier, which lies in the Yarlung Zangbo River Grand Canyon, the deepest canyon in the world. This area was the most challenging hiking area worldwide but is no longer accessible since a debris flow caused by ice avalanches originating from the Jialabailei glacier dammed the river on 16 October 2018.

Three other terminal devices were installed in remote areas of the Qilian Mountains, which stretch more than 400 km along the northeastern edge of the Qinghai-Tibet Plateau. Water resources stemming from the Qilian Mountains feed the Hexi Corridor and supply five rivers, and Qinghai Lake is the largest inland lake in China. We installed the third terminal device combined with a snow depth monitor and AWS in the Dadongshuyakou snow-covered area within the Qilian Mountains on 20 December 2019. The fourth terminal device was installed with a tree stemflow meter and AWS in the Dayekou forest area of the Qilian Mountains on 27 December 2019, and the fifth device was deployed nearby on 10 August 2020, combined with ten dendrometers to monitor tree growth.

### 4.2. Analysis of the Retrieved Data

**Figure 6:**
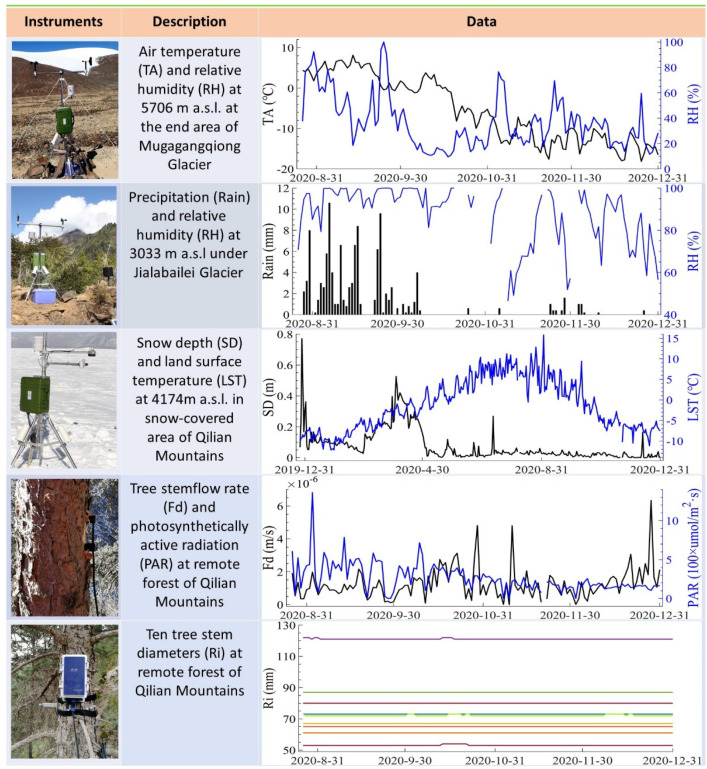
Featured instruments and observation data. The instrument column provides the environmental monitoring device deployed in the farmost areas on the TP, the description column briefly introduces the featured variables, and the data column presents the variation in these data retrieved from the surrounding area of two glaciers on the TP and remote areas in the Qilian Mountains.

From 8–26 August 2020, we activated the terminal devices installed at the five remote stations’ locations, and the devices began to transmit data, as shown in Figure 6. This period also marks the initiation of field data transmission by the satellite-enabled IoRT network from the most remote areas of the TP. Operational satellites could transmit half-hourly observations back to the data center twice a day.

The featured observations included meteorological data retrieved from the areas surrounding the Mugagangqiong and Jialabailei glaciers on the TP and snow depth, water consumption, and tree growth rate data retrieved from the Qilian Mountains in the northeastern TP. Half-hourly air temperature, wind speed, and other meteorological data retrieved from the end of the Mugagangqiong glacier, a typical continental glacier that is recognized as one of the world’s most sensitive regions to climate change, could enable detailed monitoring of the daily variability in weather conditions in the surrounding alpine desert weather.

The Jialabailei glacier is a typical marine glacier whose rapid accumulation and melting processes lead to frequent ice avalanches, some of which resulted in debris flows that dammed the Yarlung Zangbo River and created disasters. Half-hourly precipitation, humidity, and other meteorological data acquired near the glacier could aid stakeholders in the identification of damming events along the Yarlung Zangbo River, facilitating a rapid and scientifically informed responses to river-damming disasters.

The collected snow depth and surface temperature data in the Dadongshuyakou snow-covered area could reflect snow accumulation and melting processes at high altitudes in the Qilian Mountains. These data could improve our understanding of the areal water cycle. Near real-time snow depth data could also be used to identify large snowfall events and provide early warnings for traffic safety on an important highway linking Qinghai and Gansu Provinces in this area.

Furthermore, the transmitted tree stemflow rate, photosynthetically active radiation and tree stem diameter data could reflect tree evapotranspiration, photosynthesis, and growth processes, respectively, in near-real time in the remote Dayekou forest area of the Qilian Mountains. In the past, the collection of these types of data was expensive and laborious.

However, it is important to note that due to the reduction in transmission time caused by topographical blocking, ~19% of the data obtained from stations in canyons and deep trenches could not be transmitted, which is acceptable. This situation could be significantly improved with the launch of new satellites.

## 5. Experiments and Results

### 5.1. Experimental Setup

**Figure 7:**
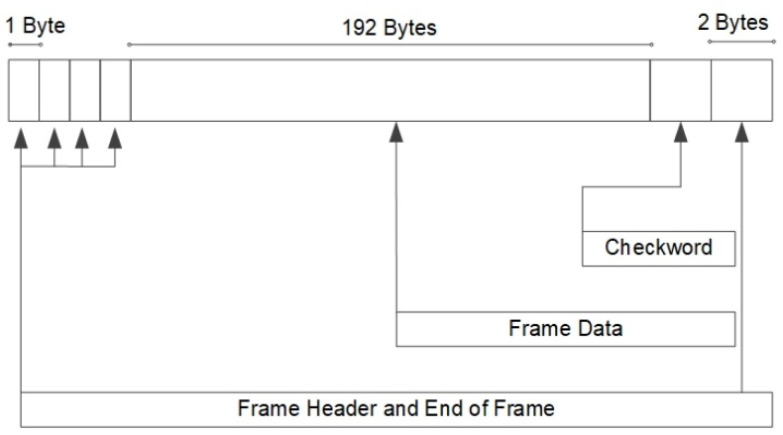
Packet structure of data transmission.

In the experiment, a performance evaluation, including an analysis of the time delay, packet loss rate, and throughput, was conducted in Wuhan, China, and the test progress was completed in accordance with the interior standards designed by Xingyun Satellite Co., Ltd., including the communication protocol between the XY-2 satellite α-stage low-mobility communication module and terminal. In the experiments, we evaluated the performance by transmitting data cached in the developed ground IoT terminals to the data center via the two Xingyun satellites. Notably, to observe the functionality and performance of the system, data of different sizes, including 54, 108, 162, 200, 324, and 400 B, were selected and cached in the ground IoT terminal in advance. Throughout the transmission process, the cached data in the ground IoT terminal were transmitted to the satellite via an uplink, transmitted to the ground gateway station located in Wuhan via a downlink, and finally transmitted to the data center via the Internet. The time delay of the system was evaluated to verify the data upload and download efficiency via satellites under a real-world scenario. The time delay was defined as the time consumption of data transferral from the ground IoT terminal to the remote data center via satellites. Because the satellite communication data bandwidth is very narrow with a maximum bandwidth of up to 200 B, it was necessary to convert the data into subdata for transmission through the satellite-enabled IoRT network. Thus, the packet creation mode was needed in the data transmission process to guarantee the success of the mission. The packet structure of the data transmission is shown in Figure 7.

### 5.2. Performance Evaluation

#### 5.2.1. System Time Delay

The packet creation mode is a method that modifies large datasets into transmittable subdata before upload to satellites. Thus, we conducted two experiments to evaluate the time delay, i.e., uploading data less than 200 B and more than 200 B.

In regard to data transmission without the packet creation mode, we set the size of a single data packet cached in the ground IoT terminal to 54, 108, and 162 B. We conducted the experiment at different times and under various weather conditions to evaluate the time delay. Table 2 summarizes the details of the field experiment. The data transmission process from the ground IoT terminal to the data center via satellites revealed low time delays of approximately 30, 31 and 37 s for the 54, 108, and 162 B data packets, respectively, and these times were obtained without any processing. Since the whole data packet was smaller than 200 B, each packet could be transmitted at once during satellite overpass. The results of this experiment demonstrated that data smaller than 200 B could be transmitted in a highly efficient manner, and the time delay was very low.

In regard to data transmission with the packet creation mode, the sizes of single data packets cached in the ground IoT terminal were 162, 324, and 486 B. Among these sizes, the 324 and 486 B packets exceeded the maximum satellite link bandwidth. Thus, we employed the packet creation mode to convert these data packets into subdata. The results are listed in Table 3. In this experiment, the 324 B packets were converted into two 162 B packets, and the corresponding time delay reached 28 s. Similarly, the 486 B packets were converted into three 162 B packets. The results similarly revealed that the packet creation mode could improve the time delay, yielding a value of 33 s. Notably, the result for the transmission of the 162 B data packets demonstrated the maximum time delay, likely because the time delay was influenced by many factors, such as the communication distance, but the time delay to transfer 162 B packets essentially agreed with the results for the transmission of the 324 and 486 B data packets, which were converted into two and three 162 B subdata packets, respectively. The results of the second experiment indicated that if the data packet exceeded 200 B, subpacket creation was needed, enabling the time consumption of transferring one data packet at a time to remain nearly the same regardless of the data amount.

The findings obtained in the above two experiments are summarized in Table 2 and Table 3. These tables indicate that to transfer one data packet at a time, transmission with the packet creation mode can maintain low time delays close to those maintained under transmission without the packet creation mode, indicating that the introduction of the packet creation mode could maintain the time delay at an acceptable level during data packet transmission. Thus, the data packet creation mode introduced into the satellite-enabled IoRT network is advantageous, as demonstrated by the results of these experiments.

#### 5.2.2. Packet Loss Rate

Additional testing was performed to experimentally validate the packet loss rate during data transmission via IoT satellites. Similarly, the experiment was conducted in two sets, i.e., data transmission with and without the packet creation mode.

Table 4 provides the packet loss rates results of the first test. According to the table, the data cached in the ground IoT terminal with a packet size of 54 B were transmitted to the data center at different test times and under various weather conditions. We compared the data received by the data center to the transmitted data to evaluate the packet loss rate and determined that the maximum packet loss rate of 9.5% occurred on 29 November 2020, at 19:02, and the minimum packet loss rate reached 0. In general, the results demonstrated that a packet loss lower than 10% could slightly affect data application.

Regarding data transmission with the packet creation mode, the transmitted data cached in the ground IoT terminal exhibited a packet size of 324 B, which were converted into two subdata packets using the packet creation mode, resulting in two 162 B subdata packets. For example, on 9 December 2020, at 8:19, the numbers of transmitted and received data packets were 54 and 52, respectively. Similarly, on 9 December 2020, at 19:00, the numbers of transmitted and received data packets reached 42 and 40, respectively, and the packet loss rates in these two tests reached 6% and 4.7%, respectively, suggesting that packet loss occurred in both packet transmission tests. Additionally, other tests, such as those conducted on 12 December 2020, at 8:19 and 13 December 2020, at 19:00, yielded similar packet loss rate results.

**Figure 8:**
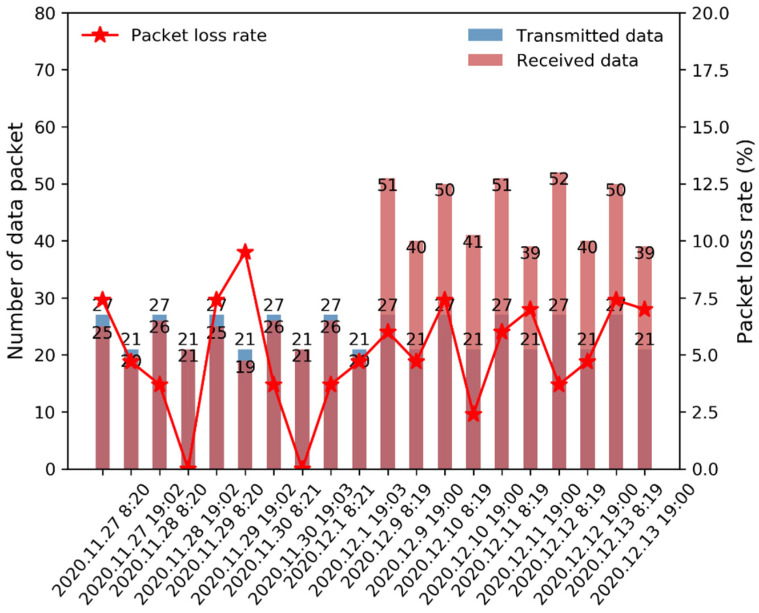
Histogram of the packet loss rate. The packet loss rate for the received data versus the transmitted data and the number of data packets versus the transmission time.

The resulting packet loss rates in these two tests are shown in Figure 8. The packet loss rates were all evidently lower than 10%, suggesting that the introduction of the packet creation mode into the satellite-enabled IoRT network could provide high-quality monitoring data retrieved from remote areas. The results listed in Table 5 are consistent with the previous results provided in Table 3, revealing an enhancement in the remote data collection, including improvements in the time delay and packet loss rate.

#### 5.2.3. Throughput

In this section, we evaluated the throughput to experimentally determine whether the satellite-enabled IoRT network functioned properly. To conduct this analysis, two experiments were performed, i.e., data transmission with and without the packet creation mode.

In regard to data transmission without the packet creation mode, we performed a field experiment and compared transmitted and received data, and the size of the transmitted data packet was 200 B. A comparison of the transmitted data versus the received data is provided in Table 6. According to the results, given a packet size of 200 B, the received data were consistent with the transmitted data, suggesting that the cached data in the ground IoT terminal could be successfully transmitted to the data center, illustrating that field data transmission via the satellite-enabled IoRT network from remote areas is feasible and that the satellite functioned properly. Note that this result was obtained based on an acceptable 10% packet loss rate, and the assessment criterion of satellite functioning was a packet loss rate of the transmitted and received data lower than 10% under the same time stamp. If this criterion is not satisfied, the satellite is not functioning properly.

Similarly, regarding data transmission with the packet creation mode, we selected data with a packet size of 400 B, and these data were thus converted into two subdata packets 200 B in size. In this experiment, 400 B data packets were selected for testing with the aim of examining the performance of cached data transmission via satellites under the maximum throughput. The results for the transmitted and received data are listed in Table 7. The transmitted data constituted a complete packet that is 400 B in size. However, the data comprised two 200 B subdata packets in the ground IoT terminal before transmission to the satellite. Thus, the received data comprised two subdata packets, and packet loss occurred in the transmission of both subdata packets. For example, on 13 December 2020, at 7:22, 125 data points were transmitted, whereas 238 data points were received. We could conclude that these results are consistent with those of the above experiment, suggesting that data exceeding the maximum throughput of the satellite link can be transmitted by employing the packet creation mode, and a highly satisfactory performance was attained. Our experimental results further demonstrated that the established satellite-enabled IoRT network could suitably retrieve data from remote areas.

## 6. Discussion

In recent years, the development of IoT satellites has burgeoned. For example, the U.S. company SpaceX is deploying a low-orbit broadband IoT constellation, i.e., Starlink, and Swarm is developing a low-orbit narrowband constellation. China has also proposed a broadband satellite constellation, namely, the Hongyun project, which has not yet been implemented, in addition to the low-orbit narrowband constellation of the Xingyun project.

In this article, we proposed a new satellite-enabled IoRT network benefitting from the Xingyun project, China’s first IoT satellite constellation, to address the challenge of retrieving monitoring data from the most remote areas of the TP lacking public networks, which could provide tremendous significance for scientific research. We implemented the proposed satellite-enabled IoRT architecture and demonstrated its application in delivering monitoring data to a data center via satellites. The innovative design implemented a newly developed ground IoT terminal allowing monitoring devices to be networked, which could be deployed in any region of interest. Because monitoring requirements are usually dynamic in terms of time and space, especially across the TP, the innovative ground IoT terminal could facilitate the flexible deployment of monitoring devices at different locations within the satellite-enabled IoRT network. Each device could transmit monitoring data to a data center, thus offering timely and effective data retrieval options to researchers.

The key aspect of the ground IoT terminal entails the integration of a LEOBIT satellite transceiver and an RR1016 datalogger. At present, the downlink transmission rate of the transceiver reaches only 2.4 kbps, and the uplink transmission rate ranges from 1.2 to 2.4, 4.8 and 9.6 kbps. Nevertheless, the terminal could satisfy the requirements of routine data transmission. Although data packet loss occurred, this phenomenon could be significantly mitigated as more Xingyun satellites are launched and integrated into the constellation. The implementation of the proposed satellite-enabled IoRT network based on the Xingyun project could trigger the large-scale deployment of automatic ground-based observation stations, heralding a new beginning for observations across the TP. Accordingly, the value of the satellite-enabled IoRT network for data retrieval from the most remote areas of the TP is very clear, as global access could enable rapid data retrieval from remote monitoring devices and could help users in a new era of environmental monitoring.

However, the transmission of data generated in high-frequency observations, such as eddy covariance and video data, still requires a broadband IoT constellation. Given that the Chinese broadband satellite constellation (i.e., the Hongyun project) has not yet been launched and that the Starlink service has not yet been introduced into the Chinese market, the development of a virtual IoT constellation through the integration of a satellite communication channel could represent an alternative solution to broaden the communication bandwidth. Consequently, we are attempting to make this objective a reality. In the future, the ideal scenario could involve the integration of satellite communication channels of all types (broad- and narrowband) to establish a global virtual IoT constellation, which would require addressing not only various technical challenges, but also various notable challenges posed by business competition and data security issues.

## 7. Conclusions

To obtain in situ monitoring data in the most remote areas of the TP, we developed an IoRT system enabled by IoT satellites launched under the Xingyun project to collect field data. A ground IoT terminal was established to operate in conjunction with an environmental monitoring device configured with a LEOBIT satellite transceiver to enable its remote IoT functionality and allow the device to transmit monitoring data to a data center via satellites.

We installed five of these newly developed ground IoT terminals across the TP to monitor the temperature and wind speed in the Mugagangqiong glacier area, precipitation (rain) and relative humidity in the Jialabailei glacier area, and snow depth, land surface temperature, tree stemflow rate, photosynthetically activated radiation, and stem diameters of ten trees in the Qilian Mountains since 2020. We evaluate the performance of the satellite-enabled IoRT system in terms of the time delay, packet loss rate, and throughput. The results indicated that the performance of transmitting cached data as packets smaller than 200 B in size was basically consistent with that of transmitting cached data as packets exceeding 200 B in size (200 B is the maximum satellite bandwidth), and the packet creation mode demonstrated its advantages in transmitting cached data over 200 B in size within the satellite-enabled IoRT network. The successful application of the satellite-enabled IoRT network represents an important step toward the ubiquitous application of environmental monitoring across the TP, which could play an undeniably important role in advancing the state of scientific research on the TP.

## Figures and Tables

**Table 1:** The power consumption of LEOBIT satellite transceiver.

Working Mode	Description	Minimum Value	Maximum Value	Unit
Terminal sleep	LEOBIT is deactivated	30	50	mA
Terminal wake-up	LEOBIT is activated	180	190	mA
Sending uplink instructions.	LEOBIT is activated	400	410	mA

**Table 2:** System time delays (without the packet creation mode).

Transmitted Time	Received Time	Weather	Data Size	Number of Transmitted Packets	Number of Received Packets	Time Delay
2020.12.7 8:21:30	2020.12.7 8:22:00	Rain	54 B	20	18	30 s
2020.12.7 19:02:34	2020.12.7 19:03:05	Rain	108 B	20	19	31 s
2020.12.8 8:21:32	2020.12.8 8:22:09	Overcast	162 B	20	20	37 s

**Table 3:** System time delay (with the packet creation mode).

Transmitted Time	Received Time	Weather	Data Size	Number of Transmitted Packets	Number of Received Packets	Time Delay
2020.12.6 8:21:40	2020.12.6 8:22:15	Rain	162 B	20	18	35 s
2020.12.6 19:02:31	2020.12.6 19:02:59	Rain	324 B	20	18	28 s
2020.12.6 19:40:05	2020.12.6 19:40:38	Rain	486 B	20	19	33 s

**Table 4:** Packet loss rate (without the packet creation mode).

Test Time	Weather	Data Size	Number of Transmitted Packets	Number of Received Packets	Packet Loss Rate
2020.11.27 8:20	Rain	54 B	27	25	7.4%
2020.11.27 19:02	Rain	54 B	21	20	4.7%
2020.11.28 8:20	Rain	54 B	27	26	3.7%
2020.11.28 19:02	Rain	54 B	21	21	0
2020.11.29 8:20	Overcast	54 B	27	25	7.4%
2020.11.29 19:02	Overcast	54 B	21	19	9.5%
2020.11.30 8:21	Overcast	54 B	27	26	3.7%
2020.11.30 19:03	Overcast	54 B	21	21	0
2020.12.1 8:21	Overcast	54 B	27	26	3.7%
2020.12.1 19:03	Overcast	54 B	21	20	4.7%

**Table 5:** Packet loss rate (with the packet creation mode).

Test Time	Weather	Data Size	Number of Transmitted Packets	Number of Received Packets	Packet Loss Rate
2020.12.9 8:19	Sunny	324 B	54	51	6%
2020.12.9 19:00	Sunny	324 B	42	40	4.7%
2020.12.10 8:19	Sunny	324 B	54	50	7.4%
2020.12.10 19:00	Sunny	324 B	42	41	2.4%
2020.12.11 8:19	Sunny	324 B	54	51	6%
2020.12.11 19:00	Sunny	324 B	42	39	7%
2020.12.12 8:19	Overcast	324 B	54	52	3.7%
2020.12.12 19:00	Overcast	324 B	42	40	4.7%
2020.12.13 8:19	Overcast	324 B	54	50	7.4%
2020.12.13 19:00	Overcast	324 B	42	39	7%

**Table 6:** Throughput (without the packet creation mode).

Test Time	Weather	Data Size	Number of Transmitted Packets	Number of Received Packets	Is the Satellite Functioning Properly?
2020.12.12 7:22	Sunny	200 B	120	113	Yes
2020.12.12 8:21	Sunny	200 B	220	210	Yes
2020.12.12 19:03	Sunny	200 B	185	176	Yes
2020.12.12 19:40	Sunny	200 B	280	265	Yes

**Table 7:** Throughput (with the packet creation mode).

Test Time	Weather	Data Size	Number of Transmitted Packets	Received Data	Is the Satellite Functioning Properly?
2020.12.13 7:22	Overcast	400 B	250	238	Yes
2020.12.13 8:21	Overcast	400 B	460	445	Yes
2020.12.13 19:03	Overcast	400 B	374	355	Yes
2020.12.13 19:40	Overcast	400 B	552	520	Yes

## Data Availability

The data presented in this study are available on request from the corresponding author.
